# Tooth Wear

**DOI:** 10.1155/2012/731085

**Published:** 2012-03-29

**Authors:** Ridwaan Omar, Anders Johansson, Ann-Katrin Johansson, Gunnar E. Carlsson

**Affiliations:** ^1^Department of Restorative Sciences, Faculty of Dentistry, Kuwait University, P.O. Box 24923, Safat 13110, Kuwait; ^2^Department of Clinical Dentistry-Prosthodontics, Faculty of Medicine and Dentistry, University of Bergen, Arstadveien 17, 5009 Bergen, Norway; ^3^Department of Clinical Dentistry-Cariology, Faculty of Medicine and Dentistry, University of Bergen, Arstadveien 17, 5009 Bergen, Norway; ^4^Department of Prosthetic Dentistry/Dental Materials Science, Institute of Odontology, The Sahlgrenska Academy, University of Gothenburg, Gothenburg, P.O. Box 450, SE 405 30, Sweden

Tooth wear has for long received much attention in an anthropological context. In contrast, the dental literature had afforded it less attention—until about the mid-1990s, when reports began appearing about the very high prevalence, in children and in adolescents, of tooth wear, due in the main to dental erosion. These findings appear to have triggered a strong interest in the subject, and over the past 10 years approximately 1500 publications have appeared in the PubMed database using the search terms “tooth erosion”, “tooth abrasion”, or “tooth attrition.” This represents a 50% increase over the preceding decade in research covering the various areas of the field. This special issue seeks to highlight some of the key areas of current interest, including topics such as mechanisms of wear, diagnosis, effects arising from systemic diseases, and different aspects of the management of the worn dentition. 

The paper entitled “*Dental erosion—from past to present, and its growing importance in clinical practice*” reviews the increasingly important topic of dental erosion, with reference to its prevalence, etiology, diagnosis, clinical characteristics, influence of individual defense and lifestyle factors, and an overview of management considerations. The paper entitled “*New perspectives on tooth wear*”, from a more fundamental standpoint, addresses the different types, causes, sources and mechanisms of wear, and why worn teeth appear the way they do. The paper entitled “*Gastroesophageal reflux disease and tooth erosion*” describes the detrimental effect of gastroesophageal reflux disease on the dental hard tissues, focusing in particular on the frequently overlooked condition of “silent reflux” in the causation of tooth wear. The paper entitled “*Biologically-based restorative management of tooth wear*” emphasizes the need for a biological perspective to be taken in the various approaches to managing tooth wear. The paper entitled “*Restoration of noncarious cervical lesions: when, why, and how*” addresses the different aspects of the etiology of NCCLs and the particular challenges that such defects present when they need restoring. 

It is hoped that the contents of this special issue might provide valuable insights, to the clinician and the researcher, into the important topic of tooth wear.

*Ridwaan Omar* *Ridwaan Omar* *Anders Johansson* *Anders Johansson* *Ann-Katrin Johansson* *Ann-Katrin Johansson* *Gunnar E. Carlsson* *Gunnar E. Carlsson*

